# Dengue Fever (DF) in Pakistan

**DOI:** 10.1186/1447-056X-10-1

**Published:** 2011-02-24

**Authors:** Fridous Jahan

**Affiliations:** 1 Family Medicine Department, Aga Khan University Hospital, Stadium Road PO Box 3500, Postal Code 74800, Karachi, Pakistan

## Abstract

Dengue is a widespread mosquito-borne infection in human beings, which in recent years has become a major international public health concern. Symptomatic dengue virus infections can present with a wide range of clinical manifestations, from a mild febrile illness to a life-threatening shock syndrome. Both viral and host factors are thought to contribute to the manifestations of disease in each infected. It is important to understand its burden on health care, morbidity and mortality. Early diagnosis and suspicion of DF in primary care might reduce the complications if handled properly. We must understand the depth of the problem in terms of its transmission, clinical presentation, diagnosis, management and prevention.

## Background

The World health Organization (WHO) declares dengue and dengue hemorrhagic fever to be endemic in South Asia. WHO currently estimates there may be 50 million dengue infections worldwide every year. In 2007 alone, there were more than 890 000 reported cases of dengue in the Americas, of which 26 000 cases were Dengue Hemorrhagic Fever (DHF)[1]. The disease is now endemic in more than 100 countries in Africa, Americas, the Eastern Mediterranean, South-east Asia and the Western Pacific. South-east Asia and the Western Pacific are the most seriously affected.

Pakistan is at high risk of being hit by large epidemics because of many over crowded cities, unsafe drinking water, inadequate sanitation, large number of refugees and low vaccination coverage. These conditions promote the spread of infectious diseases and consequently every year a large number of epidemics/outbreaks occur in different parts of the country, which result in increased morbidity and mortality.

## Epidemiology

### Global Burden

Dengue virus infection is increasingly recognized as one of the world's emerging infectious diseases. About 50-100 million cases of dengue fever and 500,000 cases of Dengue Hemorrhagic Fever (DHF), resulting in around 24,000 deaths, are reported annually[1].

A pandemic of dengue began in Southeast Asia after World War II and has spread around the globe since then. In the 1980 s, DHF began a second expansion into Asia when Sri Lanka, India, and the Maldive Islands had their first major DHF epidemics.

### Local Prevalence

Pakistan first reported an epidemic of dengue fever in 1994.The epidemics in Sri Lanka and India were associated with multiple dengue virus serotypes, but DEN-3 was predominant and was genetically distinct from DEN-3 viruses previously isolated from infected persons in those countries. In Asian countries where DHF is endemic, the epidemics have become progressively larger in the last 15 years. In 2005, dengue is the most important mosquito-borne viral disease affecting humans[2].

Dengue virus is now endemic in Pakistan, circulating throughout the year with a peak incidence in the post monsoon period. Recent flood in Pakistan made the situation worse.

Dengue Surveillance Cell Sind province of Pakistan reports 1,809 suspected Dengue out of which 881 confirmed till 11^th ^October 2010 with 5 deaths while 16 confirmed cases reported in Islamabad without any mortality.

Till now 563 confirmed cases were reported at our institution since January 2010. Reported cases are usually complicated or with hemorrhagic manifestation. In primary health care the usual presentation is mild to moderate fever treated as suspected dengue fever.

Researchers have identified that co-circulation of DEN-2 and DEN-3 was responsible for the 2006 out-break in Karachi. Primary and secondary cases were seen in both groups. Cases with DHF showed marginal association with DEN-2. Introduction of a new serotype (DEN-3) and or a genotypic shift of endemic serotype (DEN-2) are the probable factors for the recent out-break of DHF in this region[3].

## Transmission

Aedes Aegypti mosquito, which generally acquires the virus while feeding on the blood of an infected person and transmit the disease to another non infected person. It is primarily a daytime feeder lives around human habitation. This mosquito rests indoors, in closets and other dark places. Outside, it rests where it is cool and shaded. The female mosquito lays her eggs in water containers in and around homes, schools and other areas in towns or villages. These eggs become adults in about 10 days. Dengue mosquitoes also breed in stored, exposed, water collection systems. The favored breeding places are: barrels, drums, jars, pots, buckets, flower vases, plant saucers, tanks, discarded bottles/tins, tires, or water coolers, and other places where rainwater collects or stored.

Dengue infection is caused by any of 4 different serotypes of the virus (DEN-1, DEN-2, DEN-3, and DEN-4). After an incubation period of 2-8 days after an infective mosquito bite, the disease usually begins with sudden onset of fever and headache.

## Clinical Features

### WHO Case definition

Dengue fever is a severe, flu-like illness that affects infants, young children and adults, but seldom causes death. The clinical features of dengue fever vary according to the age of the patient. Infants and young children may have a non-specific febrile illness with rash. Older children and adults may have either a mild febrile syndrome or the classical incapacitating disease with abrupt onset and high fever, severe headache, pain behind the eyes, muscle and joint pains, and rash[4].

Common presentations in our clinical practice, high grade fever typically accompanied by any of the following: chilliness, retro-orbicular pain, photophobia, backache, severe muscle ache (one synonym of dengue is "break-bone fever"), and joint ache, nausea, vomiting, abdominal pain. High fever may be sustained over 5-6 days. Other signs and symptoms include a generalized maculopapular rash, lymph node enlargement, hepato splenomegaly, a positive tourniquet test, petechiae, and other hemorrhagic manifestations, such as epistaxis and gastrointestinal bleeding. In some cases it started as common cold and flu like symptoms. In general, convalescence occurs spontaneously and abruptly, but it might be prolonged, sometimes taking several weeks, and may be accompanied by pronounced asthenia and depression. In DHF characteristically, the overall vascular system is damaged, vascular instability, decreased vascular integrity and platelet dysfunction resulting in bleeding from different sites[5].

Clinical presentation may vary from undifferentiated fever, classic dengue fever(DF), Dengue hemorrhagic fever(DHF) to Dengue shock syndrome(DSS). The risk of severe disease is much higher in sequential rather than primary dengue infection[6].

Necessary Criteria for DHF:

■ Fever, or recent history of acute fever

■ Hemorrhagic manifestations

■ Low platelet count (100,000/mm^3 ^or less)

■ Objective evidence of "leaky capillaries:"

■ elevated hematocrit (20% or more over baseline)

■ low albumin

■ pleural or other effusions

Grade 1 DHF: Fever and nonspecific constitutional symptoms, Positive tourniquet test is only hemorrhagic manifestation. Thrombocytopenia and rise in haematocrit level (more than 20%).

Grade 2 DHF: Grade 1 manifestations + spontaneous bleeding, circulatory failure manifested by rapid and weak pulse, narrowing of pulse pressure (20 mmHg or less) or hypotension with the presence of cold clammy skin and restlessness, Capillary relief time more than two seconds. Thrombocytopenia and rise in haematocrit level (more than 20%)

Grade 3 DHF/DSS: Signs of circulatory failure (rapid/weak pulse, narrow pulse pressure, hypotension, cold/clammy skin)

Grade 4 DHF/DSS: Profound shock (undetectable pulse and BP), abdominal pain - intense and sustained, persistent vomiting, abrupt change from fever to hypothermia, with sweating and prostration, restlessness or somnolence.

## Laboratory Tests

Usually clinical suspicion for Dengue fever is sufficient for supportive treatment. Complete blood picture may show high hematocrit, leucopenia and thrombocytopenia. Other laboratory tests include serum albumin, chest X Ray if required. A normal blood count does not rule out DF however platelet <50,000 and leukocyte count <3 might be a sign of bad prognosis.

Diagnosis of dengue fever or its complications is established by culture of the virus itself, by detection of viral DNA with use of PCR, or by serological methods. Although detection of specific IgM indicates fresh infection, a significant increase in IgG titer in paired serum samples is also sufficient for diagnosing dengue fever. Currently employed methods include capture ELISAs, immunofluorescence tests, and hemagglutination assays. Low white cell count, low platelet count, abnormal liver function test, IgM ELISA test for serologic diagnosis, IgM detecTable 5, 6 days after the onset of illness, IgG: day 14 of illness in primary and day2 in secondary infections[7].

## Pathophysiology

Studies has shown that median age of dengue patients has decreased now and younger patients may be more susceptible in the recent outbreak. Total and differential leukocyte counts and platelet count may help identify patients at risk of hemorrhage. Severity of disease depends on virus strain, pre-existing anti-dengue antibody previous infection maternal antibodies in infants, host genetics, age, secondary infections locations with two or more serotypes circulating simultaneously at high levels (hyperendemic transmission) and virus strain (genotype)[8]. Epidemic potential is dependent on viremia level, infectivity and virus serotype, DHF risk is greatest for DEN-2, followed by DEN-3, DEN-4 and DEN-1[9]. Antibody-dependent enhancement is the process in which certain strains of dengue virus, complexed with non-neutralizing antibodies, can enter a greater proportion of cells of the mononuclear lineage, thus increasing virus production. Infected monocytes release vasoactive mediators, resulting in increased vascular permeability and hemorrhagic manifestations that characterize DHF and DSS[10].

There are other febrile illness prevalent in Pakistan like other viral infections, Malaria, Enteric fever and Congo hemorrhagic fever which can cause leucopenia and thrombocytopenia worth considering during the investigation and relevant investigations are done according to the clinical presentation[11,12].

Dengue IgM is a costly investigation not freely available although it confirms the diagnosis but never changes the management.

## Management

There is no specific treatment available the management is entirely supportive like keeping body temperature below 39°C, give the patient paracetamol (not more than four times in 24 hours. Avoid Aspirin or Brufen/Ponston. Advice to drink large amounts of fluids (water, soups, milk and juices) along with the patient's normal diet. The patient should rest. Complete blood picture should be done if fever is continue for three days. Oral rehydration salt (ORS) should be started even there is no significant clinical dehydration as patient can go in rapid deterioration if dehydration commences. Primary care physician can start intravenous fluid according to the patients need it is life saving if administered on proper time[13].

Clinical manifestation of impending hemorrhage are, abdominal pain - intense and sustained, persistent vomiting, abrupt change from fever to hypothermia, with sweating and prostration, restlessness or somnolence, Thrombocytopenia <50,000, WBC <3.0, evidence of "leaky capillaries:" high hematocrit (> 20% normal), low albumin, pleural or other effusions needs urgent referral for hospitalization.

## Health education and Prevention

Primary care professionals have the potential and ability to provide comprehensive care for most patients, given adequate training, resources, and, when needed, specialist advice. General practitioners and community nurses can play a major role in health education and hygiene[14].

Effectiveness of Family physicians' use of specific communication skills in enhancing the care of the physical, mental and emotional health of both their patients and their families is essential.

Our health educational system needs to be updated regularly, the information regarding Dengue Fever to be made more generally available, the popular sources of information like newspapers and television should be used to disseminate information on a large scale[15].

Dengue mosquitoes bite during the daytime. Protection from the bite by wearing full-sleeve clothes and long dresses to cover the limbs use of repellents, mosquito coils and electric vapour mats during the daytime. Insecticide treated nets (ITNs) are available to protect young children, pregnant women, old people, in addition to others who may rest during the day. Curtains (cloth or bamboo) can also be treated with insecticide and hung at windows or doorways, to repel or kill mosquitoes. Drainage of water from desert/window air coolers when not in use, in addition to tanks, barrels, drums, and buckets. Remove all objects containing water such as plant saucers from the house. All stored water containers should be kept covered at all times. Collect and destroy discarded containers in which water collects, such as bottles, plastic bags, tins, tyres, etc.

Vector control is implemented using environmental management and chemical methods[16]. Proper solid waste disposal and improved water storage practices, including covering containers to prevent access by egg laying female mosquitoes, are encouraged through community-based programs[17]. The application of appropriate insecticides to larval habitats, particularly those used by the households, such as water storage vessels can prevent mosquito breeding for several weeks therefore these insecticides must be used periodically [18].

Recent outbreaks have shown significant mortality and morbidity in Pakistan. The best health outcome depends upon accurate diagnosis and appropriate treatment. A patient centered communication provides a more complete clinical picture which leads to improvement in health outcomes such as symptom resolution, reduced psychological distress, improvement of health and functional status, relief from pain and anxiety control.

## Conclusion

Family Physicians have a vital and active role to play in providing care, support and identifying the sign of impending hemorrhage which is serious consequences of Dengue Fever needs referral to tertiary care for intravenous fluid replacement, platelet transfusion along with supportive care. Family practice also has opportunity for research-based evidence on Dengue fever more interventional research is required in community to eradicate this problem.

On-going public awareness campaign need to be strengthened and vigorous campaign need to be initiated at all levels. Family doctors in primary health care setting have an opportunity not only give the best possible supportive care to their patients but also educate them regarding the spread of Dengue fever and vector control.

## Competing interests

The author declares that they have no competing interests.
